# Elevated levels of plasma D-dimer predict a worse outcome in patients with nasopharyngeal carcinoma

**DOI:** 10.1186/1471-2407-14-583

**Published:** 2014-08-10

**Authors:** Wen-Hui Chen, Lin-Quan Tang, Feng-Wei Wang, Chang-Peng Li, Xiao-Peng Tian, Xiao-Xia Huang, Shi-Juan Mai, Yi-Ji Liao, Hai-Xia Deng, Qiu-Yan Chen, Huai Liu, Lu Zhang, Shan-Shan Guo, Li-Ting Liu, Shu-Mei Yan, Chao-Feng Li, Jing-Ping Zhang, Qing Liu, Xue-Wen Liu, Li-Zhi Liu, Hai-Qiang Mai, Mu-Sheng Zeng, Dan Xie

**Affiliations:** State Key Laboratory of Oncology in South China; Collaborative Innovation Centre for Cancer Medicine, Sun Yat-sen University Cancer Center, Guangzhou, 510060 P. R. China; Department of Nasopharyngeal Carcinoma, Sun Yat-sen University Cancer Center, Guangzhou, 510060 P. R. China; Department of Pathology, Sun Yat-sen University Cancer Center, Guangzhou, 510060 P. R. China; Department of Information Technology, Sun Yat-sen University Cancer Center, Guangzhou, 510060 P. R. China; Department of Clinical Laboratory, Sun Yat-sen University Cancer Center, Guangzhou, 510060 P. R. China; Department of Statistics and Epidemiology, Sun Yat-sen University Cancer Center, Guangzhou, 510060 P. R. China; Imaging Diagnostic and Interventional Centre, Sun Yat-sen University Cancer Center, Guangzhou, 510060 P. R. China

**Keywords:** Nasopharyngeal carcinoma, D-dimer, Survival

## Abstract

**Background:**

Hemostatic alterations occur during the development of cancer. Plasma D-dimer is a hypercoagulability and fibrinolytic system marker that is increased in patients with various solid tumours. The aim of this study was to evaluate the hemostatic status of nasopharyngeal carcinoma (NPC) patients by assessing plasma D-dimer levels to investigate its value as a prognostic marker.

**Methods:**

We retrospectively analysed 717 patients with nasopharyngeal carcinoma, and we applied Cox regression and log-rank tests to assess the association of D-dimer levels with disease-free survival (DFS), distant metastasis-free survival (DMFS), and overall survival (OS). D-dimer levels were measured using a quantitative D-dimer latex agglutination assay.

**Results:**

Using the 3rd quartile values (0.8 μg/L) as the optimal cut-offs, we found that patients with high D-dimer levels have a shorter 3-year DFS, (79%, 95%CI (73.1–84.9)) vs. (69%, 95%CI (59.2–78.8)), DMFS (87%, 95%CI (83.1–90.9)) vs. (77%, 95%CI (69.2–84.8)), and overall survival (82%, 95%CI (76.1–87.9)) vs. (76%, 95%CI (66.2–85.8)). Multivariate analysis revealed that pre-treatment D-dimer levels and EBV DNA were significant independent factors for DFS, DMFS, and OS in NPC patients. Subgroup analyses indicated that the plasma D-dimer levels could effectively stratify patient prognosis for early cancer, advanced stage cancer, and patients with EBV DNA ≥4000 copies/ml.

**Conclusions:**

High D-dimer levels were associated with poor disease-free survival, distant metastasis-free survival, overall survival, and increased risk of mortality in NPC patients. Prospective trials are required to assess the prognostic value of D-dimer levels.

**Electronic supplementary material:**

The online version of this article (doi:10.1186/1471-2407-14-583) contains supplementary material, which is available to authorized users.

## Background

Nasopharyngeal carcinoma (NPC) is one of the most common malignancies in southern China, southeastern Asia, and northern Africa. The lowest prevalence of NPC is found in white populations from Europe and the United States [1–3]. Radiotherapy regimens are currently the primary treatment strategy for NPC patients. Although the TNM staging system [4] is currently the most powerful prognostic factor for NPC, patients with the same stage and similar treatment regimens have variable clinical outcomes. The expression of several specific biological markers have been used to provide additional prognostic information for NPC patients, including epidermal growth factor receptor [5], serum lactate dehydrogenase (LDH) [6], C-reactive protein (CRP) [7], and plasma Epstein-Barr virus DNA (EBV DNA) [8]. Several studies have investigated pre-treatment EBV DNA, which is used for disease monitoring and prognosis prediction in the clinic [8–10].

Several studies have suggested an association between a more aggressive cancer phenotype and hypercoagulability [11, 12]. Interestingly, in the absence of venous thromboembolism (VTE), the systemic activation of blood coagulation and procoagulant changes in the hemostatic system are frequently been observed in cancer patients [13, 14]. Patients with cancer and hypercoagulation have a higher risk of venous thrombosis and a poor prognosis [15]. It has been reported that many increased or decreased coagulation factors contribute to cancer growth, progression and metastasis [16]. The plasma D-dimer is a stable end product of fibrin degradation, and it is a useful biomarker for predicting venous thromboembolism (VTE) in cancer patients [17]. Increased D-dimer levels are related to tumour stage, tumour prognosis and lymph node involvement. Additionally, D-dimer levels are a negative prognostic indicator for several malignancies, including breast [18], colorectal [19], lung [20], and prostate cancer [21]. Elevated D-dimer levels may reflect multifactorial interactions between carcinoma growth and the hemostatic-fibrinolytic system in malignancy. However, the clinical significance of D-dimer in nasopharyngeal carcinoma has not been established.

The purpose of this study was to analyse the prognostic value of plasma D-dimer levels in patients with NPC and evaluate the correlation between pre-treatment plasma D-dimer levels and clinical-pathological parameters. The results of our study will help predict NPC progression in patients and provide information for further treatment.

## Methods

### Patient selection

The principal inclusion criteria were as follows: (1) biopsy-proven primary NPC, no radiotherapy, chemotherapy, or oncologic surgery history, and an ECOG of 0 to 2; (2) patient age ≥18 years; (3) adequate hematologic, renal, and hepatic function (white blood cell count of ≥ 4000/μ L, platelet count of ≥100000/UL, serum creatinine clearance ≥ 50 mL/min, total serum bilirubin concentration < 1.5 mg/dL); and (4) available follow-up data. The following exclusion criteria were used for our study: (1) patients who had a history of venous thrombosis or anticoagulation therapy within 3 months before treatment; (2) patients with previous or coexisting cancer other than NPC; (3) pregnancy and lactation; or (4) stroke or neurosurgery within 6 months.

Between January 2008 and December 2011, a total of 717 consecutive non-distant-metastatic NPC patients (average age of 47, ranging from 29 to 71 years old) were enrolled at Sun Yat-sen University Cancer Center (Guangzhou, China). During the same time period, 126 healthy volunteers (average age of 45, ranging from 19 to 75 years old) who submitted to D-dimer testing as part of a routine physical examination in our hospital were enrolled as the control group. This retrospective study was approved by the Clinical Research Ethics Committee of the Sun Yat-sen University Cancer Center, and all the participants provided written informed consent before treatment.

The routine staging patient work-up included the following clinical examinations of the head and neck region: magnetic resonance imaging scans from the suprasellar cistern to the collarbone, fibre optic nasopharyngoscopy, chest radiography, abdominal sonography, whole-body bone scan or whole body FDG PET/CT. All patients were restaged according to the seventh American Joint Committee on Cancer (AJCC) TNM staging manual. All clinical records and magnetic resonance images were independently reviewed by 2 radiologists (X.W.L and L.Z.L.) to minimise heterogeneity in restaging. Before treatment, the following baseline clinical data were collected from the medical records and information system at the study institute: sex, age, WBC counts, neutrophil counts, haemoglobin (HGB), platelet counts, LDH, CRP, hereditary NPC, smoking status, PS by Eastern Cooperative Oncology Group (ECOG), and the presence of concurrent diseases, such as cardiovascular disease, diabetes and chronic hepatitis.

#### Treatment delivery

One hundred and sixty-seven (23.3%) patients were treated with conventional two-dimensional (2D-CRT) or three-dimensional conformal radiotherapy (3D-CRT), and the remaining 550 (76.7%) patients were treated with intensity-modulated radiotherapy (IMRT). Details of the radiotherapy techniques used at the Sun Yat-sen University Cancer Center were reported in a previous study [22, 23]. Six hundred and forty (91.3%) patients with stage II–IV disease received concurrent platinum-based chemotherapy.

A stratified multi-therapeutic protocol was used to treat patients. Radiation therapy alone was used for stage I disease, and radiation with concurrent platinum-based chemotherapy was used to treat stage II disease [24]. Concurrent chemoradiotherapy, with or without neoadjuvant or adjuvant chemotherapy, was used for advanced-stage disease (stages III and IV). Neoadjuvant or adjuvant chemotherapy consisted of cisplatin with 5-fluorouracil or cisplatin with taxane administered every 3 weeks for 2 or 3 cycles [25]. The concurrent chemotherapy regimen consisted of cisplatin given on weeks 1, 4, and 7 of RT, or weekly cisplatin. All the patients at the study institution were treated according to the principle of treatment for NPC patients at Sun Yat-sen University Cancer Center, Guangzhou, China.

### D-dimer evaluation

A 3 mL fasting blood sample was collected before treatment of NPC patients and on the day of physical examination for the healthy volunteers. The sample was processed within 3 hours of collection, and the plasma was stored at −70 to −80°C until analysis. D-dimer values were measured by a latex-enhanced immunoturbidimetric assay (Sekisui Medical Co., Ltd., Tokyo, Japan) using a Sysmex CA 7000 (Sysmex Corp., Kobe, Japan) analyser in the clinical hospital laboratory. The results were obtained using a standard curve prepared according to the manufacturer’s instructions, and the inter-assay imprecision (coefficient of variation) was < 10%.

### EBV DNA, VCA-IgA, and EA-IgA measurement

As described in previous studies [26–28], patient plasma EBV DNA concentrations were routinely measured by q-PCR before treatment. A cut-off level of 4000 copies/ml was chosen to define low and high EBV DNA levels because this threshold has previously been shown to be prognostic in NPC patients using the same measurement system [10, 29]. EBV-specific VCA/IgA antibodies and EBV-specific EA/IgA antibodies were measured using a previously described immunoenzymatic assay [30].

#### Clinical outcomes assessment and patient follow-up

The primary endpoint of our study was disease-free survival (DFS). The secondary endpoints were distant metastasis-free survival (DMFS) and overall survival (OS). We calculated DFS from the date of the first NPC diagnosis to the date of the first relapse at any site, death from any cause or the date of the last follow-up visit. DMFS was calculated from the date of the first NPC diagnosis to the date of distant relapse or patient censoring at the date of the last follow-up. OS was calculated from the date of the first NPC diagnosis to the date of death from any cause or patient censoring at the date of the last follow-up. After treatment was completed, patients were evaluated at 3-month intervals for the first 3 years and every 6 months thereafter.

### Statistical analysis

The characteristics of the patients were divided into quartiles and described by median values and the 25th-75th percentiles (due to non-normally distributed continuous variables). Mann–Whitney U-tests were used to compare continuous variables in different subgroups. The Spearman correlation test was used to examine the association between D-dimer levels and other variables. Survival curves were calculated by the Kaplan-Meier method, and univariate analyses were performed using the log-rank test. Variables that reached a p-value of ≤ 0.05 in the univariate analysis were entered into multivariate analyses. All statistical calculations were performed using SPSS 17.0 for Windows (Chicago, IL), and a P < 0.05 was considered statistically significant.

## Results

### Baseline characteristics and distribution of D-dimer level in the study population and subgroups

**Figure 1:**
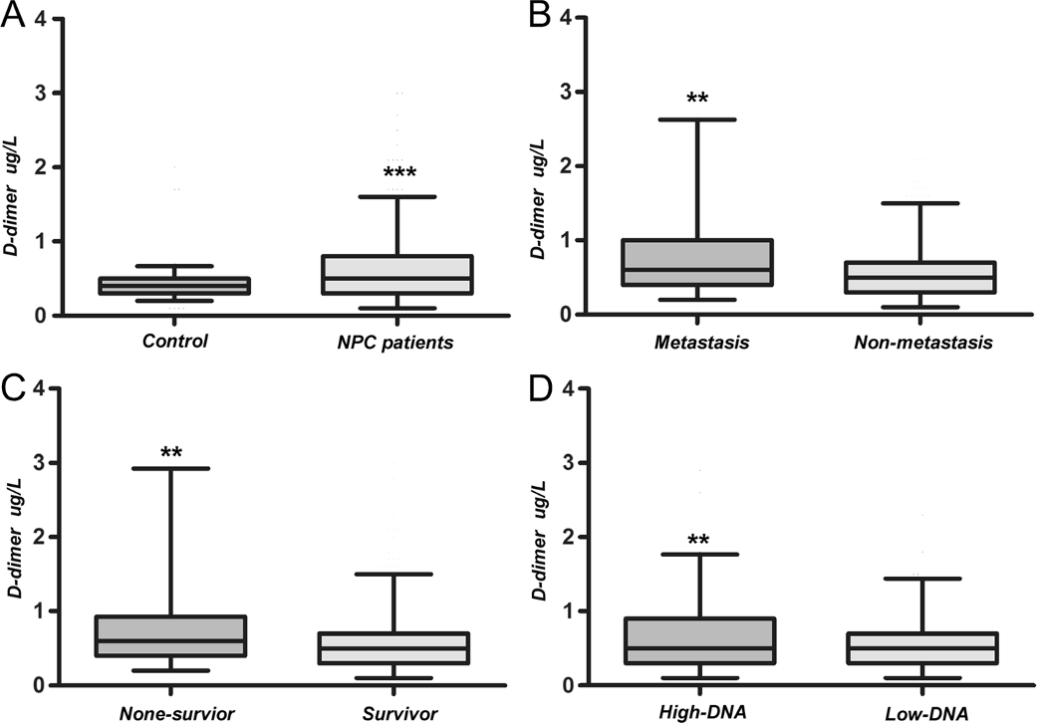
**Plasma D-dimer levels are expressed as the median and 5%-95% percentile in patients with nasopharyngeal carcinoma according to different variables. (A)** Healthy volunteers versus nasopharyngeal carcinoma patients; **(B)** Nasopharyngeal carcinoma with distant metastasis after treatment versus non-distant metastasis (P = 0.002); **(C)** Non-survivors versus survivors (P = 0.002); **(D)** High DNA (≥4000 copies/ml) versus low DNA (<4000 copies/ml) (P = 0.002).

The pretreatment characteristics of the 717 NPC patients are listed in Table 1. The median follow-up was 31 months (IQR, 24–42). Forty-eight patients developed locoregional recurrences, and 85 patients developed distant metastases. Of these patients, 16 patients had both local and distant metastases. The plasma D-dimer level was higher in NPC patients than healthy volunteers (P < 0.001, Figure 1), with the values of 0.50 (25th-75th percentile: 0.3-0.8) and 0.4 ug/mL (25th-75th percentile: 0.3-0.5), respectively. The median D-dimer level was higher in patients with distant metastasis compared to patients without distant metastasis (P = 0.002, Figure 1), with values of 0.60 (25th-75th percentile: 0.4-1.0) and 0.50 (25th-75th percentile: 0.3-0.7), respectively. The D-dimer level was higher in patients with plasma EBVDNA ≥ 4000 copies than in patients with EBVDNA < 4000 copies (P = 0.002, Figure 1), with values of 0.50 (25th-75th percentile: 0.3-0.9) and 0.50 (25th-75th percentile: 0.3-0.7), respectively. Patients who died during the observation period had significantly higher D-dimer levels at baseline than patients who were alive at the end of the study or the last follow-up: 0.6 (25th-75th percentile: 0.4-0.925) and 0.5 (25th-75th percentile: 0.3-0.7), respectively (P = 0.002, Figure 1). A Spearman correlation analysis further demonstrated that plasma D-dimer levels correlated with age, serum CRP level, LDH level, EBV DNA level, tumour TNM stage, and distant metastasis (Additional file 1: Table S1).

**Table 1:** **Patient demographics and clinical characteristics**

Characteristic	Median (25th-75th percentile) or No. (%)	Median (25th-75th percentile) or No. (%)
	NPC patients (n = 717)	Control (n = 126)
Age at study entry	47 (39–47)	45 (39–53)
Sex		
Male	533 (74.3)	102 (81)
Female	184 (25.7)	24 (19.0)
D-dimer	0.50 ug/mL (0.3-0.8)	0.40 ug/mL (0.3-0.5)
Histology, WHO type		
III	690 (96.2)	
II	27 (3.8)	
ECOG		
0-1	712 (99.3)	
2	5 (0.7)	
Clinical stage		
I	16 (2.2)	
II	80 (11.2)	
III	416 (58.0)	
IV	205 (28.6)	
Tumour stage		
T1	54 (7.5)	
T2	128 (17.9)	
T3	367 (51.2)	
T4	168 (23.4)	
Node stage		
N0	112 (15.6)	
N1	273 (38.1)	
N2	277 (38.6)	
N3	55 (7.7)	
Treatment		
Radiotherapy	75 (10.5)	
Chemotherapy and radiotherapy	642 (89.5)	
Radiotherapy technique		
2DRT/3DCRT	167 (23.3)	
IMRT	550 (76.7)	
EBVDNA		
Low DNA	411 (57.3)	
High DNA	306 (42.7)	
VCA-IgA		
<1:80	223 (31.1)	
≥1:80	494 (68.9)	
EA-IgA		
<1:10	315 (43.9)	
≥1:10	402 (56.1)	
LDH, U/L		
< 167	353 (49.2)	
≥167	364 (50.8)	
CRP, mg/L		
< 1.61	359 (50.1)	
≥1.61	358 (49.9)	
WBC, 109/L		
< 6.7	362 (50.5)	
≥6.7	355 (49.5)	
Neutrophil, 109/L		
< 4.0	359 (50.2)	
≥4.0	358 (49.8)	
HGB, g/L		
< 143.8	359 (50.1)	
≥ 143.8	358 (49.9)	
PLT, 109/L		
< 227	359 (50.1)	
≥227	358 (49.9)	
Smoking		
yes	259 (63.9)	
no	458 (36.1)	
Chronic HBV Infection		
yes	57 (7.9)	
no	660 (92.1)	
Cardiovascular disease		
yes	61 (8.5)	
no	656 (91.5)	
Diabetes mellitus		
yes	22 (3.1)	
no	695 (96.9)	
Family history of NPC		
yes	61 (8.5)	
no	656 (91.5)	
Median follow-up (months)	31 (24–44)	
Outcome features		
Progression		
yes	118 (16.5)	
no	599 (83.5)	
Distant metastasis		
yes	85 (11.9)	
no	632 (88.1)	
Localregional recurrence		
yes	48 (6.7)	
no	669 (93.3)	
Deaths		
yes	94 (13.1)	
no	623 (86.9)	

*Abbreviations*: *ECOG*  Eastern Cooperative Oncology Group, *2DRT*  two-dimensional radiotherapy, *3DCRT*  three-dimensional conformal radiotherapy, *IMRT*  intensity-modulated radiotherapy, Low DNA denotes a low EBV DNA level of <4000 copies/ml, High DNA denotes a high EBV DNA level of ≥4000 copies/ml, *VCA*  viral capsid antigen. *IgA*  immunoglobulin A, *EA*  early antigen, *LDH*  Serum Lactate Dehydrogenase Levels, *CRP*  high-sensitivity C-reactive protein, *WBC*  White blood cell, *HGB*  haemoglobin, *PLT*  platelet, *NPC*  nasopharyngeal carcinoma.

### D-dimer levels and probability of survival

For the Kaplan-Meier analysis, patients were categorised into the following four groups according to their D-dimer levels: the1st group (D-dimer levels ranging from minimum to 1st quartile of D-dimer levels in the total cohort population: 0.0-0.3 μg/mL), 2nd group (D-dimer levels from 1st quartile to 2nd quartile: 0.3-0.5 μg/mL), 3rd group (D-dimer levels from 2nd quartile to 3rd quartile: 0.5-0.8 μg/mL) and 4th group (D-dimer levels from 3rd quartile to maximum: 0.8-37.2 μg/mL). Additional file 1: Figure S1 shows the Kaplan-Meier estimates for DFS, DMFS and OS according to D-dimer levels. Elevated D-dimer levels were significantly associated with shorter DFS, DMFS and OS (log-rank trend test: P < 0.001).

**Figure 2:**
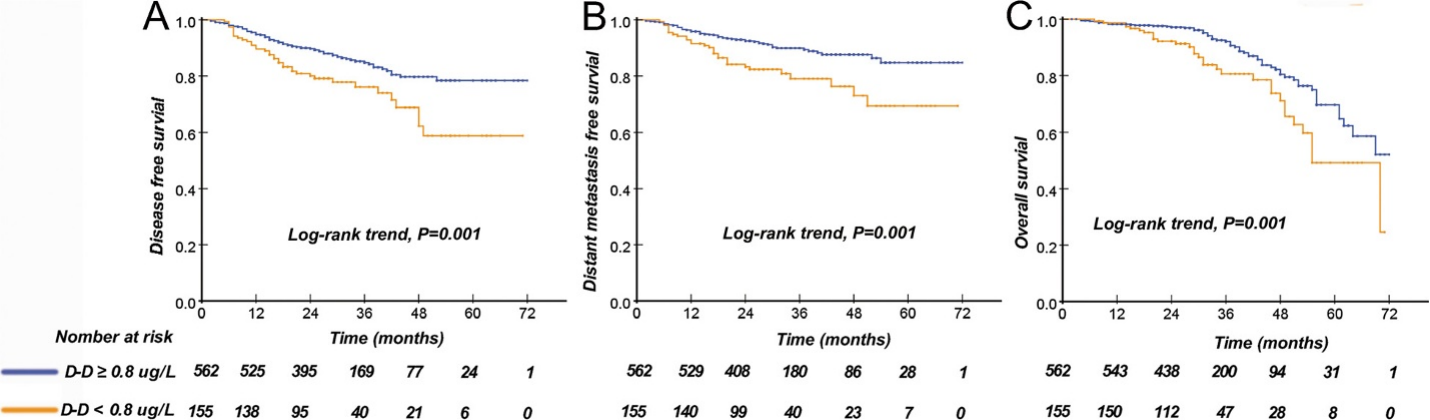
**Kaplan-Meier curves of disease-free survival (A), distant metastasis-free survival (B), and overall survival (C) of the entire NPC population according to pre-treatment D-dimer levels (n = 717).**

Using the 1st group as a reference, the unadjusted hazard ratio (HR) for DFS, DMFS, and OS of the top quartiles were 2.26 (95% CI, 1.38-3.70), 2.99 (95% CI, 1.63-5.48), and 3.06 (95% CI, 1.70-5.51), respectively. A similar trend of adjusted HR was observed for DFS, DMFS and OS. HRs and 95% CIs comparing quartiles of D-dimer for disease-free, distant metastasis and overall survival are detailed in Additional file 1: Table S2. The patients were divided into two groups based on whether the D-dimer level was above or below the top quartiles (0.8 μg/L). Patients with high D-dimer levels had a shorter 3-year DFS, (79%, 95%CI (73.1–84.9)) vs. (69%, 95%CI (59.2–78.8)), DMFS (87%, 95%CI (83.1–90.9)) vs. (77%, 95%CI (69.2–84.8)), and overall survival (82%, 95%CI (76.1–87.9)) vs. (76%, 95%CI (66.2–85.8)) (Figure 2).

### Univariate and multivariate analyses of prognostic factors

As shown in Table 2, using 0.8 ug/L as a cut-off value, the hazard ratios (HRs) and 95% CIs estimated from Cox regression models indicated that the D-dimer level was strongly associated with DFS, DMFS, and OS (HR, 1.88; 95% CI, 1.28 to 2.77), (HR, 2.16; 95%CI, 1.38 to 3.36) and (HR, 1.98; 95%CI, 1.29 to 3.04), respectively. In addition to elevated D-dimer levels, the UICC TNM classification, high EBV DNA and elevated CRP levels (1.61 mg/L) were significantly associated with DFS, DMFS, and OS.

**Table 2:** **Univariate Cox proportional hazards regression analysis**

Characteristic	DFS (n = 717)			DMFS (n = 717)			OS (n = 717)		
	HR	95% CI	***P***	HR	95% CI	***P***	HR	95% CI	***P***
Age, (≥47 vs. < 47 years)	0.73	0.51 to 1.06	0.100	0.87	0.56 to 1.33	0.517	0.85	0.56 to 1.29	0.444
Male vs. Female	1.08	0.71 to 1.65	0.726	1.48	0.86 to 2.54	0.162	0.92	0.64 to 1.65	0.922
Histology, WHO type (III vs. II)	0.95	0.42 to 2.53	0.949	0.69	0.28 to 1.70	0.689	1.78	0.56 to 5.62	0.328
ECOG (2 vs. 0–1)	1.10	0.15 to 7.85	0.927	1.05	0.36 to 6.53	0.596	1.25	0.14 to 8.99	0.824
**Clinical stage (III-IV) vs. (I-II)**	**3.05**	**1.34 to 6.94**	**0.008**	**4.43**	**1.40 to 14.01**	**0.011**	**2.56**	**1.04 to 6.31**	**0.041**
**Tumour stage (T3-4 vs. T1-2)**	**1.47**	**0.93 to 2.31**	**0.100**	**1.65**	**0.94 to 2.88**	**0.08**	**1.32**	**0.80 to 2.18**	**0.286**
**Node stage (N2-3 vs. N0-1)**	**1.72**	**1.19 to 2.48**	**0.004**	**2.19**	**1.40 to 3.42**	**0.001**	**1.75**	**1.16 to 2.66**	**0.008**
**Chemoradiotherapy (yes v no)**	**1.94**	**0.90 to 4.17**	**0.089**	**1.58**	**0.69 to 3.63**	**0.279**	**1.59**	**0.70 to 3.64**	**0.27**
**IMRT vs. 2DRT/3DCRT**	**0.55**	**0.38 to 0.80**	**0.002**	**0.66**	**0.42 to 1.04**	**0.071**	**0.88**	**0.57 to 1.34**	**0.537**
High DNA Vs. Low DNA	2.97	2.02 to 4.36	< 0.001	3.69	2.30 to 5.91	< 0.001	3.50	2.23 to 5.50	< 0.001
**CRP, mg/L (≥1.61vs. < 1.61)**	**1.43**	**0.99 to 2.06**	**0.055**	**1.83**	**1.18 to 2.84**	**0.007**	**1.87**	**1.23 to 2.85**	**0.003**
VCA-IgA (≥1:80 vs. < 1:80)	1.39	0.91 to 2.13	0.127	1.32	0.81 to 2.16	0.267	0.95	0.64 to 1.61	0.952
EA-IgA (≥1:10 vs. < 1:10)	1.23	0.85 to 1.77	0.282	1.17	0.76 to 1.81	0.477	0.99	0.66 to 1.50	0.977
**LDH, U/L (≥167 vs. < 167)**	**1.41**	**0.98 to 2.04**	**0.064**	**1.67**	**1.07 to 2.59**	**0.002**	**1.38**	**0.92 to 2.08**	**0.125**
**D-dimer, ug/L (≥0.8 vs. < 0.8)**	**1.88**	**1.28 to 2.77**	**0.001**	**2.16**	**1.38 to 3.36**	**0.001**	**1.98**	**1.29 to 3.04**	**0.002**
WBC, 109/L (≥6.7 vs. < 6.7)	1.06	0.90 to 1.25	0.753	1.17	0.76 to 1.9	0.481	1.08	0.72 to 1.62	0.709
Neutrophil, 109/L (≥4.1 vs. < 4.1)	1.09	0.76 to 1.56	0.658	1.03	0.67 to 1.57	0.9	1.24	0.82 to 1.87	0.308
HGB, g/l (≥143.8 vs. < 143.8)	0.87	0.61 to 1.25	0.463	0.82	0.53 to 1.25	0.35	0.77	0.51 to 1.16	0.207
PLT, 109/L (≥227 vs. < 227)	1.13	0.79 to 1.62	0.511	1.08	0.71 to 1.66	0.712	1.23	0.82 to 1.84	0.33
**Smoking (yes vs. no)**	**1.27**	**0.88 to 1.84**	**0.196**	**1.59**	**1.04 to 2.44**	**0.033**	**1.22**	**0.81 to 1.84**	**0.346**
Chronic HBV Infection (yes vs. no)	1.00	0.84 to 1.18	0.953	1.02	0.84 to 1.23	0.881	0.97	0.80 to 1.18	0.969
cardiovascular disease (yes v no)	1.16	0.61 to 2.22	0.653	1.28	0.62 to 2.67	0.5	1.64	0.79 to 3.41	0.184
Diabetes mellitus (yes v no)	0.94	0.85 to 1.05	0.286	0.97	0.87 to 1.08	0.592	0.94	0.84 to1.05	0.248
family history of NPC (yes v no)	1.14	0.61 to 2.11	0.686	1.13	0.55 to 2.35	0.735	1.14	0.92 to 1.41	0.241

*Abbreviations*: *ECOG*  Eastern Cooperative Oncology Group, *2DRT*  two-dimensional radiotherapy, *3DCRT*  three-dimensional conformal radiotherapy, *IMRT*  intensity-modulated radiotherapy, Low DNA denotes a low EBV DNA level of <4000 copies/ml, High DNA denotes a high EBV DNA level of ≥4000 copies/ml, *VCA*  viral capsid antigen. *IgA*  immunoglobulin A, *EA*  early antigen, *LDH*  Serum Lactate Dehydrogenase Levels, *CRP*  high-sensitivity C-reactive protein, *WBC*  White blood cell, *HGB*  haemoglobin, *PLT*  platelet, *NPC*  nasopharyngeal carcinoma. *DFS*  disease-free survival, *DMFS*  metastasis-free survival, *OS*  overall survival.

Multivariate analysis indicated that elevated D-dimer levels were a highly significant predictor for DFS, DMFS, and OS, independent of EBV DNA level, LDH level, CRP levels, UICC TNM staging, smoking status, and treatment type (Table 3).

**Table 3:** **Multivariate Cox proportional hazards regression analysis**

Characteristic	DFS (n = 717)			DMFS (n = 717)			OS (n = 717)		
	HR	95% CI	***P***	HR	95% CI	***P***	HR	95% CI	***P***
Tumour stage (T3-4 vs. T1-2)	1.23	0.76 to 1.99	0.395	1. 36	0.76 to 2.45	0.305	1.04	0.61 to 1.79	0.88
Node stage (N2-3 vs. N0-1)	1.14	0.76 to 1.71	0.516	1.43	0.88 to 2.33	0.148	1.08	0.68 to 1.72	0.756
Chemoradiotherapy (yes vs. no)	1.23	0.55 to 2.74	0.621	0.84	0.40 to 2.02	0.7	0.89	0.36 to 2.18	0.793
IMRT vs. 2DRT/3DCRT	0.62	0.42 to 0.90	0.013	0.76	0.48 to 1.21	0.246	0.99	0.64 to 1.52	0.954
High DNA vs. Low DNA	2.41	1.59 to 3.64	< 0.001	2.77	1.66 to 4.62	< 0.001	3.06	1.87 to 5.02	< 0.001
CRP, mg/L (≥1.61 vs. < 1.61)	1.14	0.78 to 1.68	0.495	1.35	0.85 to 2.14	0.21	1.48	0.95 to 231	0.082
LDH, U/L (≥167 vs. < 167)	1.22	0.84 to 1.77	0.290	1.35	0.86 to 2.11	0.189	1.23	0.81 to 1.86	0.34
D-dimer, ug/L (≥0.8 vs. < 0.8)	1.60	1.08 to 2.37	0.020	1.79	1.13 to 2.82	0.013	1.58	1.02 to 2.45	0.043
Smoking (yes vs. no)	1.26	0.87 to 1.82	0.216	1.58	1.03 to 2.43	0.036	1.35	0.89 to 2.06	0.159

*Abbreviations:* *ECOG*  Eastern Cooperative Oncology Group, *2DRT*  two-dimensional radiotherapy, *3DCRT*  three-dimensional conformal radiotherapy, *IMRT*  intensity-modulated radiotherapy, Low DNA denotes a low EBV DNA level of <4000 copies/ml, High DNA denotes a high EBV DNA level of ≥4000 copies/ml, *LDH*  Serum Lactate Dehydrogenase Levels, *CRP*  high-sensitivity C-reactive protein, *DFS*  disease-free survival, *DMFS*  metastasis-free survival, *OS*  overall survival.

### Prognostic significance of D-dimer within the UICC TNM classification, and patients with high or low EBVDNA level

**Figure 3:**
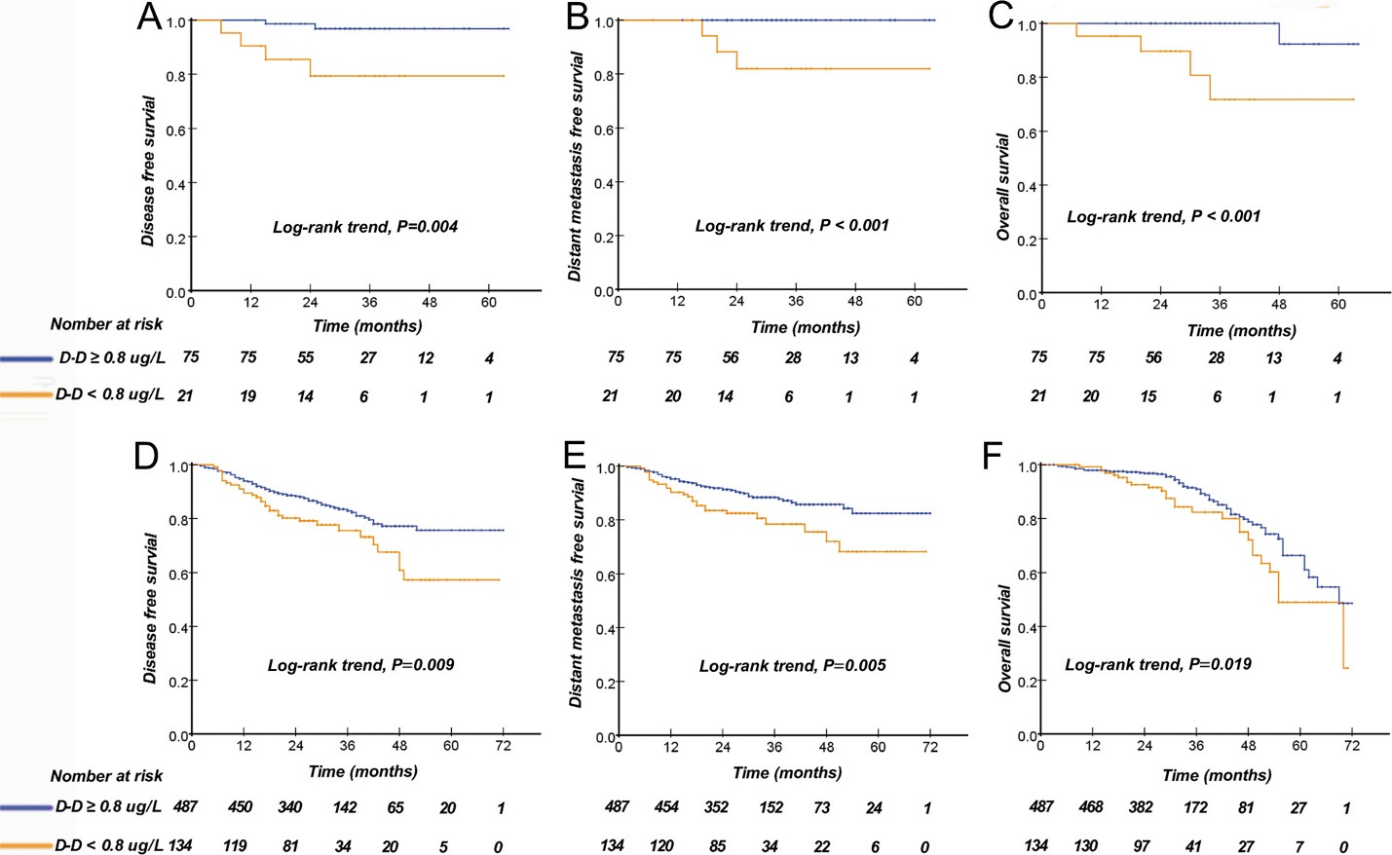
**Kaplan-Meier curves of disease-free survival (A), distant metastasis-free survival (B), and overall survival (C) of NPC patients with early stage (I + II, n = 96), and disease-free survival (D), distant metastasis-free survival (E), and overall survival (F) of NPC patients with advanced stage (III + IV, (n = 621)), according to pre-treatment D-dimer levels.**

Given the independent prognostic significance of elevated D-dimer levels in NPC patients, we evaluated the discrimination power of elevated D-dimer levels in early-stage and advanced-stage patients. Compared to patients with low D-dimer levels, patients with early-stage and advanced-stage disease with elevated D-dimer levels had shorter DFS, DMFS, and OS (Figure 3).

**Figure 4:**
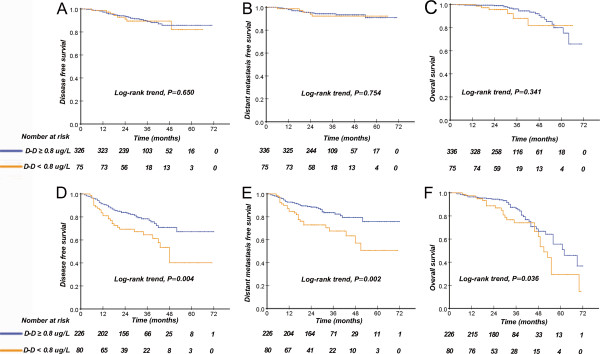
**Kaplan-Meier curves of disease-free survival (A), distant metastasis-free survival (B), and overall survival (C) of NPC patients with plasma EBV DNA < 4000c copies/ml (n = 411), and disease-free survival (D), distant metastasis-free survival (E), and overall survival (F) of NPC patients with the plasma EBV DNA ≥ 4000 copies/ml, according to pre-treatment D-dimer levels.**

Consistent with previous reports [10, 29], we chose 4000 copies/ml to define the low and high pre-treatment EBVDNA level. In subgroup analysis, patients in the high EBV DNA subgroup with elevated D-dimer levels had a worse DFS, DMFS, and OS compared with the patients with low D-dimer levels. For patients with low EBV DNA levels, there was no difference in DFS, DMFS, and OS between the patients with low or high D-dimer levels (Figure 4).

## Discussion

To our knowledge, this is the first large-scale cohort study examining the association between coagulation and survival in nasopharyngeal carcinoma patients. The activation of coagulation in cancer patients is widely implicated in both tumour progression and the development of thrombosis [31]. High levels of plasma D-dimer have been associated with poor prognosis in several malignant diseases, including lung pancreas, prostate, gastric, colorectal, and breast cancer [19, 20, 32, 33]. Recently, Liu et al. [32] demonstrated that elevated plasma D-dimer was correlated with depth of invasion, lymph node metastasis, peritoneal dissemination, distant metastasis, tumour size, TNM stage, and worse overall survival in gastric cancer. Interestingly, according to our findings, the D-dimer plasma level is a marker of endogenous fibrinolysis that can be used to evaluate hypercoagulability, and it has both clinical and prognostic significance in nasopharyngeal carcinoma patients. With a median follow-up of 3 years, the HR of DFS, DMFS, and OS in cancer patients with the highest D-dimer levels in the total study population (D-dimer levels ranging from 3rd quartile to the maximum level) was two-fold higher than patients with D-dimer levels ranging from the minimum to the 1st quartile. Using the 3rd quartile (0.8 μg/mL) as the final cut-off, multivariate analysis revealed that pre-treatment D-dimer levels were a significant, independent prognostic factor for predicting recurrence, distant metastasis, and death. This effect was independent of EBV DNA level, LDH level, CRP levels, UICC TNM staging, smoking status, and treatment type (Table 3).

Plasma EBV DNA has been demonstrated to be correlated with tumour burden [34], TNM stage [35], response to chemoradiotherapy [36–38], and survival in NPC patients [8, 10, 29]. Plasma EBV DNA is now a useful biomaker for the clinical management of NPC patients, and it is considered the most attractive potential biomarker [37]. However, for NPC patients with EBV DNA ≥4000 copies/ml, patients with D-dimer ≥ 0.8 μg/L still have a shorter DFS, DMFS, and OS compared to patients with D-dimer < 0.8 μg/L. However, this result does not apply to patients with EBV DNA < 4000 copies/ml. Although the magnitude of the predictive value of EBV DNA was superior to that of D-dimer, it is not sufficient to use plasma EBV DNA alone for prognostic stratification due to the heterogeneity of NPC patients. Notably, patients in the high EBV DNA subgroup with high plasma D-dimer levels had a worse disease-free survival, distant metastasis-free survival, and overall survival. These results indicate that EBV DNA alone is insufficient to complement the TNM classification, and plasma D-dimer levels represent a complementary marker to EBV DNA leves for the prediction of NPC patient prognosis.

Although the mechanism underlying D-dimer-mediated NPC progression of remains unknown, it is likely due to different D-dimer signalling pathways or biological behaviours. The association between the D-dimer levels and NPC progression may be explained by abnormalities in haemostasis and fibrinolysis during tumour pathogenesis. The activation of coagulation is the result of increased tissue factor expression, which leads to fibrin deposition. Tumour cells also express tissue factors, and they might contribute to a variety of pathological processes, such as VTE, metastatic spread, tumour growth, and tumour angiogenesis [39].

Interestingly, elevated D-dimer levels still predicted a poorer DFS, DMFS, and OS for early and advanced stage NPC patients in both high and low EBV DNA subgroups, independent of TNM staging (Figure 3). Recent advancements in NPC patient classification and NPC molecular alterations have been made, including microRNA signatures [40] and the NPC-SVM classifier [41]. However, these developments require expensive and complicated procedures, and rapid clinical implementation was difficult to achieve in a short time. Plasma D-dimer levels are established, routinely measured blood-based parameters that are reproducibly detected without additional laborious efforts before use in clinical applications. These findings have great clinical relevance because D-dimer subgroups with different prognoses in defined TNM stages indicate the need for individual treatment for NPC patients in the future. The association between pre-treatment D-dimer levels and TNM stage may be helpful for screening NPC patients. If a patient displays an abnormally high D-dimer level before treatment, they may require more intensive systemic approaches to improve the treatment outcome.

However, there are still some limitations to the present study, such as the single D-dimer measurements recorded from a single centre. Another limitation of this study is that we neglected to collect data for venous thromboembolism (VTE) events. High D-dimer levels have previously been reported to predict VTE in cancer patients [42, 43], and both D-dimer and VTE are negative prognostic factors for cancer patients [33]. The third limitation was the short follow-up time, and additional patient data will be reported after the the 5 year follow-up.

## Conclusions

In summary, we demonstrated that pre-treatment D-dimer levels can reflect hypercoagulability in NPC patients. Our results suggest that D-dimer is a promising prognostic biomarker associated with disease-free, distant metastasis-free and overall survival. D-dimers represent a widely available potential biomarker, and the assays to detect D-dimer levels have been sufficiently validated. D-dimers should be considered for routine testing in NPC patients. A prospective study is needed to explore this area, and our results should be validated using other datasets.

## Electronic supplementary material

Additional file 1: Figure S1: Cumulative probability of disease-free survival (DFS), distant metastasis-free survival (DMFS), and overall survival in the total study population (n=717). (1) Patients in the 1st group, with D-dimer levels ranging from minimum to 1st quartile (0.00 to 0.3 μg/mL) levels in the total study population, are compared to (2) those with D-dimer levels in the 2nd group, with levels between the 1st and 2nd quartiles [0.30 to 0.50μg/mL], (3) and those in the 3rd group, with levels between the 2nd and 3rd quartiles [0.50 to 0.80μg/mL], and (4) the 4th group, with D-dimer levels ranging from the 3rd quartile to maximum [0.80to 37.2μg/mL] of D-dimer levels in the study population. The total numbers of patients with D-dimer levels in the 1st, 2nd, 3rd and 4th groups at study institute were 212, 202, 148 and 155, respectively. **Table S1.** D-dimer Relationships. **Table S2.** Prognostic value of D-dimer for DFS, DMFS, and OS by Quartiles. (DOCX 54 KB)
