# Supplementary material for: Elevated levels of plasma D-dimer predict a worse outcome in patients with nasopharyngeal carcinoma
Source: BMC Cancer. 2014 Aug 10;14:583. doi: 10.1186/1471-2407-14-583 (PMC4242497; doi:10.1186/1471-2407-14-583)
Supplement: Supplementary file 1 — Additional file 1: Figure S1: Cumulative probability of disease-free survival (DFS), distant metastasis-free survival (DMFS), and overall survival in the total study population (n=717). (1) Patients in the 1st group, with D-dimer levels ranging from minimum to 1st quartile (0.00 to 0.3 μg/mL) levels in the total study population, are compared to (2) those with D-dimer levels in the 2nd group, with levels between the 1st and 2nd quartiles [0.30 to 0.50μg/mL], (3) and those in the 3rd group, with levels between the 2nd and 3rd quartiles [0.50 to 0.80μg/mL], and (4) the 4th group, with D-dimer levels ranging from the 3rd quartile to maximum [0.80to 37.2μg/mL] of D-dimer levels in the study population. The total numbers of patients with D-dimer levels in the 1st, 2nd, 3rd and 4th groups at study institute were 212, 202, 148 and 155, respectively. Table S1. D-dimer Relationships. Table S2. Prognostic value of D-dimer for DFS, DMFS, and OS by Quartiles. (DOCX 54 KB) [file 12885_2014_5015_MOESM1_ESM.docx]

**Additional file 1**

| **Table S1. D-dimer Relationships** | | |
| --- | --- | --- |
| Variable | Spearman correlation coefficient | ***P*** |
| **Age** | **0.123** | **0.001** |
| Sex (male/female) | -0.088 | 0.019 |
| Histology, WHO type (II/III) | 0.069 | 0.065 |
| ECOG (0/1/2) | 0.038 | 0.315 |
| **Clinical stage (1/2/3/4)** | **0.139** | **< 0.001** |
| **Tumour stage (1/2/3/4)** | **0.13** | **< 0.001** |
| Node stage (0/1/2/3) | 0.03 | 0.423 |
| **EBV DNA (copies/ml)** | **0.106** | **0.005** |
| VCA-IgA | 0.037 | 0.325 |
| EA-IgA | 0.038 | 0.314 |
| **LDH (U/L)** | **0.106** | **0.004** |
| **CRP, mg/L** | **0.195** | **< 0.001** |
| WBC (109/L) | 0.044 | 0.245 |
| Neutrophil (109/L) | 0.05 | 0.183 |
| **HGB (g/L)** | **-0.205** | **< 0.001** |
| PLT（109/L） | 0.03 | 0.419 |
| Smoking (yes/no) | -0.01 | 0.799 |
| Chronic HBV Infection (yes/no) | -0.012 | 0.749 |
| Cardiovascular disease (yes/no) | 0.023 | 0.534 |
| Diabetes mellitus (yes/no) | 0.016 | 0.664 |
| family history of NPC (yes/no) | -0.033 | 0.38 |
| **Distant metastasis** | **0.113** | **0.002** |
| Localregional recurrence | -0.009 | 0.816 |

Abbreviations: ECOG = Eastern Cooperative Oncology Group, LDH= Serum Lactate Dehydrogenase Levels; CRP=high-sensitivity C-reactive protein; HGB= haemoglobin; PLT= platelet counts

| **Table S2. Prognostic value of D-dimer for DFS, DMFS, and OS by Quartiles** | | | | |
| --- | --- | --- | --- | --- |
| D-dimer, ug/L | Number of Patients ( n=717) | | | |
|  | unadjusted HR | *P* | adjusted HR | *P* |
| DFS |  |  |  |  |
| < 0.3 | Reference |  | Reference |  |
| ≥0.3 to 0.5 | 1.425 (0.86-2.36) | 0.17 | 1.39 (0.84-2.31) | 0.206 |
| ≥0.5 to 0.8 | 1.20 (0.67-2.16)) | 0.541 | 1.24 (0.68-2.24) | 0.481 |
| ≥0.8 | 2.26 (1.38-3.70) | < 0.001 | 2.34 (1.36-3.70) | 0.002 |
| DMFS |  |  |  |  |
| < 0.3 | Reference |  | Reference |  |
| ≥0.3 to 0.5 | 1.69 (0.90-3.18) | 0.104 | 1.68 (0.89-3.17) | 0.11 |
| ≥0.5 to 0.8 | 1.58 (078-3.78) | 0.205 | 1.61 (0.79-3.27) | 0.19 |
| ≥0.8 | 2.99 (1.63-5.48) | < 0.001 | 3.00 (1.63-5.56) | < 0.001 |
| OS |  |  |  |  |
| < 0.3 | Reference |  | Reference |  |
| ≥0.3 to 0.5 | 1.85 (1.01-3.40) | 0.047 | 1.79 (0.97-3.31) | 0.064 |
| ≥0.5 to 0.8 | 2.10 (1.08-4.09） | 0.028 | 2.14 (1.10-4.18) | 0.025 |
| ≥0.8 | 3.06 (1.70-5.51) | < 0.001 | 2.80 (1.54-5.09) | 0.001 |

Abbreviations: DFS= disease-free survival; DMFS=distant metastasis-free survival; OS= overall survival

P* values compare risk events for patients in the first quartile.

Adjusted HR were adjusted for age (≥47 years vs.<47 years), sex, N stage (N2-3 vs. N0-1), T stage (T3-4 vs.T1-2), LDH (≥167 U/L vs.<167 U/L), smoking status (yes vs. no), concurrent cardiovascular disease (yes vs. no), diabetes (yes vs. no), and chronic hepatitis (yes vs. no).
